# Hypofractionation in Unresectable Stage II–III NSCLC: A Systematic Technical Review and Practical Recommendations

**DOI:** 10.3390/cancers18182997

**Published:** 2026-09-16

**Authors:** Gabriela Antelo, Miriam Núñez-Fernández, Josep Carreras, Nuria Farre-Bernardo, Paloma Sosa Fajardo, Inmaculada Romero-Palomar, Nuria Rodríguez de Dios, Arturo Navarro-Martin

**Affiliations:** 1 Radiation Oncology Department, Hospital Clinic of Barcelona, 08036 Barcelona, Spain; antelo@clinic.cat (G.A.); jcarreras@clinic.cat (J.C.); 2 Radiation Oncology Department, Institut Català d’Oncologia, 08908 Barcelona, Spain; mnfernandez@iconcologia.net; 3 Radiation Oncology Department, Hospital Sant Pau, 08041 Barcelona, Spain; nfarre@santpau.cat; 4 Radiation Oncology Department, Hospital Virgen del Rocío, 41013 Sevilla, Spain; paloma.sosa.sspa@juntadeandalucia.es; 5 Gregorio Marañón General University Hospital Library, 28007 Madrid, Spain; inmaculada.romero@salud.madrid.org; 6 Hospital Clinico Universitario de Santiago de Compostela, 15706 Santiago de Compostela, Spain; nuria.rodriguez.de.dios@sergas.es

**Keywords:** hypofractionated radiotherapy, unresectable Stage II/III NSCLC, radical radiotherapy

## Abstract

**Background:** Concurrent chemoradiotherapy (cCRT) followed by 1 year of consolidation durvalumab remains the standard of care for unresectable stage III non-small cell lung cancer (NSCLC), yet long-term survival is still limited and around 20% of patients present locoregional relapse. Hypofractionation may improve the therapeutic ratio by increasing the biologically effective dose (BED), shortening overall treatment time (OTT) and potentially mitigating radiation-induced lymphopenia. **Objective:** To identify which patients with unresectable stage II–III NSCLC may benefit most from radical-intent hypofractionated radiotherapy (HypoRT), with or without systemic therapy, and to define the technical aspects, contouring margins, and dosimetric requirements necessary for the safe use of hypofractionated schedule. **Methods:** A systematic search of PubMed, EMBASE, CENTRAL and Web of Science (January 2010–January 2024, English language) was performed following PRISMA principles. Eligible studies included unresectable NSCLC treated with a BED10 > 40 Gy, allowing induction systemic or targeted therapy; reviews were included for context. Records were screened by six independent reviewers. Studies were classified as non-photon vs. photon, and photon studies were further divided into non-concurrent and concurrent strategies. Clinical outcomes (dose, fractionation, biologically effective Dose for alpha/beta:10 (BED10), equivalent dose of 2 Gy/fraction (EQD2), overall treatment time (OTT), follow-up (FU), median progression-free survival (mPFS), median Overall Survival (mOS), local control (LC), grade ≥ 3 toxicity (G3) and technical variables (immobilization, simulation, GTV imaging, CTV/PTV margins, delivery, image-guided radiotherapy (IGRT), organ at risk (OAR) constraints) were extracted. **Results:** Of 340 records (170 duplicates), 26 studies including stage II–III disease were analyzed (1155 patients): 1 prospective cohort, 5 phase I, 12 phase II, 1 phase III and 2 phase I/II dose-escalation trials, 2 carbon-ion and 3 proton series. Of these, 13 were photon-based studies with contemporary simulation techniques. Two clinically distinct scenarios emerged. Group A (non-concurrent platinum-based strategies; 166 patients with modern 4DCT simulation) showed wide variability, ranging from a simultaneous-integrated-boost regimen to 75 Gy with 0% grade ≥ 3 toxicity and mPFS 40 months to a chemotherapy-driven schedule with 70% grade 4 chemo-related toxicity. Only one phase III trial was identified, reporting a 2-year local control of 85.8%. Group B (concurrent strategies; nine of the 14 platinum-based studies retrieved, namely those using modern simulation techniques) achieved a median BED of 89.7 Gy to the highest dose-volume, median of OS 20 months and toxicity GIII ≥ ranging 10–20%, with a median study-level grade 5 toxicity of 5.5% (range 0–17.8%; 34 fatal events among 431 evaluable patients, 7.9%), rising to 20.8% (11/53) in the most escalated randomized arm and 22.2% (2/9) in the highest dose-escalation cohort; adaptive SABR boost to 70 Gy/15 fr delivered high local control but clinically meaningful fatal toxicity. Fatal toxicity was confined to schedules exceeding BED10 100 Gy delivered to the whole tumor volume with concurrent platinum, whereas an equivalent BED restricted to a metabolically defined subvolume without concurrent chemotherapy produced no grade ≥3 events. **Conclusions:** Radical-intent hypofractionation is feasible across both unfit and fit unresectable stage II–III NSCLC populations, but the randomized evidence remains scarce and heterogeneous. Benefit appears most consistent when high BED is delivered with strict normal-tissue sparing in carefully selected patients using modern 4DCT-based simulation, IGRT and intensity-modulated delivery. The pattern of fatal toxicity with aggressive concurrent systemic treatments and dose escalation schedules argues for caution and for prospective trials with harmonized minimum technical standards.

## Simple Summary

Patients with stage II–III non-small cell lung cancer (NSCLC) who are unfit for concurrent chemoradiotherapy have limited curative options. Hypofractionated radiotherapy delivers higher doses per fraction over a shorter overall treatment time, offering a practical alternative to conventional schedules. In this review, we summarize prospective and retrospective evidence on hypofractionated regimens in stage II–III NSCLC, focusing on local control, survival, and toxicity outcomes with photon-based and other modern radiotherapy techniques. We considered two main patient groups: those unfit for chemotherapy and those fit for chemotherapy. Our findings suggest that carefully selected hypofractionated protocols can provide competitive tumor control with acceptable toxicity, while improving convenience and resource use in routine clinical practice.

## 1. Introduction

The standard of care (SoC) for unresectable stage III non-small cell lung cancer (NSCLC) is the combination of concurrent chemoradiotherapy (cCRT) followed by adjuvant durvalumab for 1 year [1].

HypoRT in lung cancer represents a therapeutic strategy with the potential to improve these results. This potential improvement is based on three main mechanisms: an increase in the biologically effective dose delivered, which translates into greater local control; a reduction in the overall treatment time (OTT), decreasing the likelihood of accelerated repopulation; and, by shortening the overall treatment time, a reduction in the risk of radiotherapy-induced lymphopenia.

The International Association for the Study of Lung Cancer (IASLC) recently published a systematic review of prospective studies using accelerated HypoRT with or without concurrent chemotherapy, concluding that the randomized evidence is scarce and recommending its use with caution and, when appropriate, using advanced radiation techniques [2].

However, even in this recent systematic review, some aspects remain insufficiently defined, including which patients are most likely to benefit from this approach, the minimum technical requirements for the safe delivery of hypofractionated regimens, and consensus guidelines for target volume delineation and dose constraints when using moderately hypofractionated schedules.

In this systematic review, we examine which patient subpopulations might benefit most from a hypofractionation strategy, based on the available evidence, and identify the minimum technical and dosimetric recommendations necessary to support this approach according to the current literature.

## 2. Material and Methods

A systematic search was performed in PubMed, EMBASE, CENTRAL (Cochrane Central Register of Controlled Trials), and Web of Science to identify relevant studies published between January 2010 and January 2024, in English language. The search strategy included the following terms and their combinations: “non-small cell lung carcinoma,” “NSCLC,” “stage III,” “unresectable,” “inoperable,” “radiation dose hypofractionation,” and “hypofractionation.” (Supplementary Table S1).

The inclusion criteria were unresectable NSCLC, BED10 greater than 40 Gy, English language, dose per fraction > 2 Gy and lower than 8 Gy, and induction with systemic therapy or targeted therapy. Reviews were allowed. In vitro studies, non-human studies, conference abstracts, and papers not available online were excluded.

Fractionation was classified by dose per fraction (d) using the thresholds adopted by contemporary consensus documents [3,4]: conventional fractionation, d = 1.8–2.2 Gy; moderate hypofractionation, d > 2.2–4.0 Gy; and ultra-hypofractionation (SABR-like), d > 4.0–8.0 Gy. Schedules exceeding 8 Gy per fraction were excluded because the validity of the linear-quadratic model becomes uncertain in that range.

**Figure 1:**
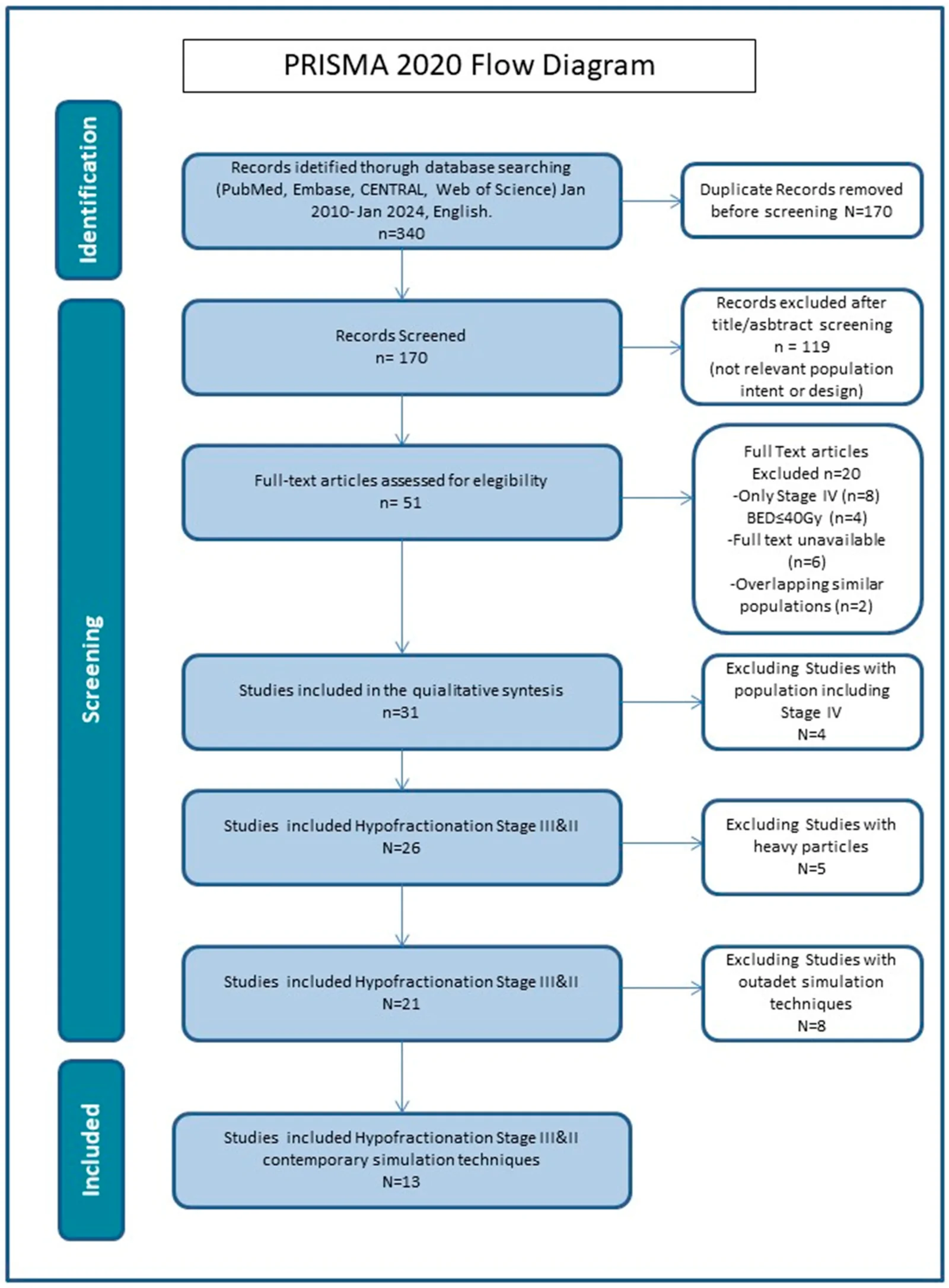
Prisma diagram.

The results of the bibliographic search were reviewed by six independent reviewers (NF, NR, GA, MN, PS, AN). In case of uncertainty regarding article inclusion, the articles were categorized as potentially eligible and underwent a second round of review. If doubt persisted after the second round, each article was discussed individually until consensus was reached, as shown in Figure 1.

Included articles were manually reviewed and classified into two categories: non-photon studies and photon studies. Photon studies, which represented the most commonly used technique, were further divided into non-concurrent strategies and concurrent strategies.

Variables were recorded according to two main objectives: clinical outcomes and technical characteristics. The primary clinical outcome variables included the following: total administered dose, fractionation schedule, BED10, equivalent dose in 2 Gy per fraction (EQD2, assuming α/β = 10), OTT (in weeks), median FU, mPFs, median overall survival, and G ≥ 3 toxicity. The technical variables collected included the following: type of immobilization used, simulation technique, imaging modality used for gross tumor volume (GTV) delineation (e.g., PET/CT), expansion margins for clinical target volume (CTV) and planning target volume (PTV), treatment delivery technique, image-guided radiotherapy (IGRT) technique, and OAR constraints applied. This systematic review was registered in PROSPERO (CRD42024519535).

The methodological quality of the selected studies was evaluated using the National Institutes of Health (NIH/NHLBI) study-quality assessment tools, which are widely used in evidence reviews.

## 3. Results

The search identified 340 articles, of which 170 were duplicates; the remaining records were reviewed by the panel of independent reviewers. Thirty-one studies were analyzed: 2 prospective cohorts, 11 phase I, 2 phase I/II studies, 15 phase II and 1 phase III. From them, three phase I studies included a stage IV population [5,6,7] and two included stage IV patients [8,9]. The only published phase III trial (Iyengar P, 2021) [10] was restricted to stage II and III disease.

Stage III was the most studied stage for hypofractionation (30 studies), followed by stage II (18 studies), as would be expected. In total, 1373 patients were treated with a hypofractionated regimen. Focusing on studies that included only stage II and III disease (excluding those including stage IV), 1155 patients were distributed across 26 studies: 2 prospective cohort, 8 phase I, 13 phase II, 2 phase I/II dose-escalation trials and 1 phase III.

Of these 26 studies, those by Takahashi et al. (2015) [11] and Saitoh et al. (2018) [12] used carbon-ion particles, and three used protons (Hoppe et al. 2022 [13], Contreras et al. 2022 [14], Gomez et al. 2013 [15]. Thus, among the 21 photon-based studies, the authors focused on routinely used contemporary techniques and selected studies that used 4DCT or slow CT for treatment simulation, for a total of 13 studies including a population of 644 patients.

Within the photon group—the most frequent irradiation method—two possible populations and treatment approaches emerged:

Group A—Non-concurrent platinum-based strategies.

Group B—Concurrent platinum-based strategies. Patients considered fit for concurrency in whom the goal is to improve local control and shorten the OTT of radiotherapy. Some authors suggest that patients whose OTT is shorter than 4 weeks have a lower probability of lymphopenia [16].

Quantitative synthesis was considered and not undertaken. Local control was reported as an actuarial estimate in only 5 of the 13 photon studies, and the remainder used crude in-field failure proportions, local progression within the planning target volume, RECIST locoregional response, freedom from intrathoracic progression, or did not report a local endpoint; grade ≥ 3 toxicity was reported as event counts rather than as the number of affected patients in 5 studies; toxicity was graded on four different scales with differing attribution rules. Pooling under these conditions would produce summary estimates whose precision would not reflect the underlying evidence.

Group A: Non-concurrent platinum-based strategies

In total, four studies were selected—one phase I, two phase II, and one phase III—comprising 166 patients with unresectable stage II and III disease treated without concurrent therapy, employing modern simulation techniques and photon-based radiotherapy (Table 1).

The phase I trial published by Ohri et al. [17], with 25 patients, enrolled a population, not candidate, for chemotherapy who received induction pembrolizumab over three cycles. A PET scan was then performed: if the metabolic tumor volume exceeded 20 cc, 55 Gy in 20 fractions was delivered, and if it was ≤20 cc, 48 Gy in 20 fractions was prescribed. Treatment was consolidated with pembrolizumab for up to one year. The trial achieved a median progression-free survival (mPFS) of 26 months with 28% grade III toxicity and no grade IV or V toxicity.

Two phase II studies have been published. The study by Parisi et al. [18] used neoadjuvant and consolidative chemotherapy around radiotherapy, delivering 30 Gy in 5 fractions to the primary and 25 Gy in 5 fractions to nodal disease. In the study by Zhang et al. [19], without any systemic therapy, a dose escalation up to 75 Gy was delivered to the macroscopic tumor volume with a simultaneously integrated boost (SIB). This population achieved an mPFS of 40 months with no G3 or higher toxicity, in contrast with the 9.7 months and 70% grade IV toxicity reported by the Parisi group [18].

Finally, the only published phase III trial [10], in patients unfit for concurrent systemic therapy, included 96 patients randomized to conventional fractionation at 2 Gy/day to 60 Gy versus hypofractionation at 4 Gy/day until 60 Gy total dose. Although no significant difference in 1-year OS was observed between the two arms, at 37.7% (95% CI, 24.2–51.0%) for the hypofractionated arm vs. 44.6% (95% CI, 29.9–58.3%) for the conventional arm, local control was higher in the hypofractionation arm, although not statistically significant at 85.8% (66.2–94.5%) vs. 66.1% (40.0–83.0%); *p* = 0.34).

Group B: Concurrent platinum-based strategies, where hypofractionated RT is used to improve response

Nine concurrent studies with contemporary simulation were identified: 3 phase I studies with 61 patients (Table 2) and 6 phase II studies with 417 patients (Table 3), for a Group B total of 478 patients. Together with the 4 non-concurrent studies of Group A (166 patients), the photon cohort analyzed comprises 13 studies and 644 patients. Concurrent therapy used platinum-based regimens, the CBDCA-VNR schedule being the most frequent.

In terms of treatment strategies, the study by Roy et al. [26] used neoadjuvant chemotherapy, and Qiu et al. [23] used induction radiotherapy. In the rest of the studies, concurrency was delivered with platinum-based schedules, with a median overall treatment duration of 4.6 weeks (range 3–6). In the concurrent studies, the median BED10 delivered to the highest-dose volume was 89.7 Gy (range 59.5–115.5), corresponding to an EQD2 of 74.6 Gy (range 49.6–96.3).

Among the dose-escalation phase I studies (Table 2), the trial by Wu et al. [22] stands out as the largest, with 28 patients. They used induction with 40 Gy in 10 fractions, followed by a sequential boost to residual metabolic disease, without a treatment break. The sequential boost was escalated across three arms: 25 Gy (low-dose cohort), 30 Gy (intermediate-dose cohort), or 35 Gy (high-dose cohort), all delivered in five fractions. The primary endpoint was the maximum tolerated dose (MTD), which was not reached. Patients were simulated with 4DCT and a vacuum cushion or similar system. The initial PTV was an ITV expansion of 5 mm, and the sequential-boost PTV was the metabolic volume without margin. Only one patient received consolidation immunotherapy, and 100% completed the treatment schedule. Regarding toxicity, two patients in the low-dose cohort had G3 toxicity and two patients in the high-dose cohort had grade 5 pulmonary toxicity. These results contrast with the 2-year LC in each cohort, the best result being the high-dose cohort with 100% LC at two years and a median PFS of 10.5 months.

Among the phase II studies (Table 3), the work by Cooke et al. [25] stands out, with 107 patients treated, and as the one with the longest follow-up at 73.3 months. This study was closed prematurely due to slow accrual. Patients received at least 72 Gy, 3 Gy/fr in 24 fractions 1 fr/day. A dose escalation was performed according to dosimetric compliance, but it affected only the primary tumor volume; nodal disease was prescribed at 2.75 Gy/fraction. The escalated dose per fraction ranged from 3 Gy/fr up to 5.4 Gy/fr. The cohort was randomized into two arms according to PET uptake: in arm A, the boost volume included all pathological uptake, and in arm B, a subvolume was added to cover the pathological uptake of 50% or more of the SUVmax. All patients were treated with intensity modulation, and although they achieved 97% one-year local control in the whole-tumor group, 15% experienced grade 5 toxicity.

Finally, the study by Qiu et al. [23] is notable for the number of patients recruited as well as for being the study with one of the highest-BED achieved. They used induction radiotherapy up to 51 Gy in 17 fractions with a break of up to 90 days, after which a CT was performed and a replanning boosted the residual disease with 15–18 Gy in 5–6 fractions, reaching a total BED10 of 84.15 Gy local control at 2 y 66.5% with G3 toxicity: 24.8% and G5: 3.3%.

### 3.1. Stratification of Studies Based on BED Ranges

The thirteen photon studies are grouped by the biologically effective dose delivered to the highest-dose volume, since this is the axis along which their outcomes diverge most sharply. Four strata were defined a priori (Table 4): low, BED10 below 60 Gy; standard, 60–79.9 Gy; escalated, 80–99.9 Gy; and ablative, 100 Gy or above. Fatal toxicity in this review was concentrated in the ablative stratum, but only where the escalated dose was delivered to the whole tumor volume together with concurrent platinum (Table 4).

### 3.2. Quality of the Studies Analyzed

The Quality Assessment Tool for Case Series Studies (9 items) was applied to the single-arm phase I, phase II, phase I/II and prospective-cohort studies, and the Quality Assessment of Controlled Intervention Studies (14 items) was applied to the randomized trials (Iyengar, Roy and the PET-Boost dataset). Each item was rated as met (+), not met (−), or cannot determine/not applicable/not reported (CD/NA/NR), and an overall rating of Good, Fair or Poor was assigned by consensus.

Two structural features of this literature dominated the appraisal. First, the great majority of studies are non-comparative case series. Secondly, among the randomized trials, blinding of participants and outcome assessors was generally absent (an inherent limitation of radiotherapy–dose comparisons) so they obtain limited ratings despite adequate randomization and analysis. No study was rated Poor; the better-reported, prospectively designed trials with adequate follow-up were Ohri 2024 (SPRINT) [17], Qiu 2021 (GASTO1011) [23], Wu 2024 [22], Parisi 2019 [18], Zhang W 2015 [19], Urbanic 2018 [21] and Van Diessen 2020 [24], which achieved a Good rating. These results are summarized in Table 5 and Table 6.

### 3.3. Treatment Volumes Contouring

Across the analyzed studies, target definition was markedly heterogeneous, reflecting the wide period of time of the literature reviewed and the evolution of functional imaging. Gross tumor volume (GTV) was delineated on PET/CT in roughly half of the cohorts and on CT alone (or CT with optional PET) in the remainder. The most contemporary and particle-therapy studies consistently incorporated 18F-FDG-PET, and several recent series defined an internal GTV (iGTV) on 4DCT or end-inhalation imaging to account for respiratory motion (Table 7).

Clinical target volume (CTV) expansion from GTV was most commonly 5–8 mm for the primary tumor, with a few series using a histology-adapted margin (e.g., 8 mm for adenocarcinoma vs. 6 mm for other histologies [37]). Elective nodal irradiation (ENI) was the exception rather than the rule: most modern photon studies treated involved nodes only (GTV-N + 5 mm), whereas the carbon-ion series (Takahashi, Saitoh) deliberately included prophylactic hilar and mediastinal stations. The most aggressive ablative-boost designs reduced or eliminated the CTV margin, prescribing the boost to the residual metabolic volume with little or no expansion.

Planning target volume (PTV) margins clustered around 5 mm in studies that already used 4DCT/iGTV-based motion management, and 10–15 mm in older 3D studies that built the geometric uncertainty into the PTV. Trials reporting the most favorable therapeutic ratios were those that moved respiratory-motion compensation into an iGTV and applied a tight (≤5 mm) PTV margin, rather than a single large expansion. This pattern reinforces the case for 4DCT-based simulation and iGTV definition as a minimum technical standard. Table 4 summarizes the imaging and margin choices of each study.

### 3.4. OAR Most Frequent Restrictions

OAR reporting was inconsistent, but several dominant conventions emerged (Table 8).

For the lung volume, the combination of V20 (≤30–35%) and mean lung dose (≤16–20 Gy) was by far the most frequently applied pair, mirroring the constraints used in conventionally fractionated locally advanced NSCLC, which was the only validated reference available to trials designed across this period. Table 8 is therefore a record of practice in studies published between 2015 and 2024 and not a statement of current consensus, which was established after all of them: for a 60 Gy in 15 fraction schedule, the ASTRO Dose-Volume Histogram Compendium specifies lungs−GTV/iGTV V16.5 Gy ≤ 37%, V5 Gy ≤ 65% and a mean dose ≤ 16.5 Gy, while the 2025 French organs-at-risk update specifies lungs−ITV mean dose < 14 Gy and V15 Gy < 20%—different metrics computed on a different structure. Similarly, the spinal cord was almost universally constrained by a maximum dose of ≤45 Gy, although several hypofractionated schedules tightened this to ≤37–40 Gy; the latter practice anticipated the limits that consensus has since adopted, ≤36.5 Gy (ASTRO) or ≤39.5 Gy with a 3 mm planning organ-at-risk volume at ≤42 Gy (French update), whereas ≤45 Gy would now exceed both at 4 Gy per fraction.

Esophageal reporting was the most heterogeneous: studies variably used mean dose (≤34 Gy), maximum dose, V35–V55, or an irradiated-length constraint, reflecting the absence of any consensus on hypofractionated esophageal tolerance throughout the period in which these trials were conducted. That absence has since been partly remedied but not resolved: for the same 60 Gy in 15 fraction schedule, the two most recent consensus documents differ by more than 8 Gy in the permitted maximum dose (≤58.9 Gy versus <50.5 Gy), and the ASTRO mean-dose goal of ≤25 Gy is designated panel consensus rather than literature-derived. Within this review, the only comparative evidence on the point is internal to the PET-Boost trial, whose protocol amendment adding an esophagus + 5 mm maximum-dose objective was followed by a fall in grade 4–5 esophageal toxicity from 5 of 54 to 0 of 53 patients—a within-trial comparison that stands independently of any subsequent guideline.

Cardiac constraints, when reported, were usually expressed as V40 (≤40–50%) or a mean-dose limit, consistent with the post-RTOG 0617 emphasis on cardiac sparing. Constraints for the brachial plexus and the proximal bronchial tree were reported only by a minority of studies—chiefly the particle-therapy series and those treating central or ultracentral tumors—underscoring a reporting gap that any future prospective hypofractionation protocol should close, given the disproportionate late-toxicity risk of large fractions to these serial structures.

### 3.5. Eligibility Criteria of the Included Studies

The eligibility criteria stated in each primary report are reproduced in Table 9. Four different performance-status instruments are used across the thirteen studies, only four specify a quantitative pulmonary function threshold, no study reports a validated comorbidity index, and the two most restrictive eligibility sets in the review belong to non-concurrent studies while two concurrent studies admitted patients with ECOG 2. In this sense, it is noteworthy that the performance status requirements do not align with the treatment strategy in the expected direction: the two most restrictive eligibility sets in this review belong to non-concurrent studies, whereas two concurrent studies admitted patients with ECOG 2.

### 3.6. Systematic Synthesis of Toxicity

Grade ≥ 3 toxicity is reported by organ system in Table 10. Main toxicities such as esophageal, pulmonary, great vessels and hematologic are described. Six studies report fatal (grade 5) toxicity and five report fatal hemorrhage; the study-level and arm-level audit, with explicit denominators, is given in Supplementary Table S2. The median study-level grade 5 toxicity was 5.5% (range 0–17.8%), with 34 fatal events among 431 evaluable patients (7.9%).

Regarding hematologic toxicity, two studies suggest that the severe toxicity is chemotherapy-attributed rather than radiation-attributed: Parisi et al. report grade 4 neutropenia in 12 patients and grade 4 leucopenia in 4 patients with no severe acute radiation normal-tissue events, and both fatal events reported by Roy et al. were hyponatremia and neutropenia. Urbanic et al. excluded grade 3 hematologic toxicity and grade 3 acute esophagitis from the protocol definition of a dose-limiting toxicity.

Lymphopenia has been described only in the GASTO 1011 trial. Grade 3 lymphopenia occurred in 50 of 89 patients (56.2%) and grade 4 in a further 19 (21.3%), so 69 of 89 patients (77.5%) developed grade ≥3 lymphopenia despite a radiotherapy delivery time of 30 days. The same trial documents a physiological cost that no grading scale captures: the mean DLCO fell from 79.9% to 70.7% of predicted (*p* < 0.001), and post-break FEV1/FVC% and DLCO% were significantly associated with overall survival, with a two-year survival of 0% in patients whose FEV1/FVC% fell to 41–59%.

## 4. Discussion

This review aligns with the conclusion of the recent IASLC’s publication regarding accelerated hypofractionated radiotherapy: prospective and randomized evidence remains scarce. Moreover, the published studies are heterogeneous in stage, intent and technique, and the approach should be used with caution and with advanced radiation techniques. This review extends that publication in two ways. First, by restricting the analysis to radical-intent treatment of unresectable stage II–III disease and excluding palliative and stage IV populations, we obtain a more homogeneous and clinically actionable cohort of 644 patients across 13 studies. Second, by separating patients into two pragmatic clinical scenarios patients in whom concurrent strategies are not an option (Group A) and patients in whom concurrent platinum-based strategies with hypofractionation are used to intensify treatment and shorten OTT (Group B), we can frame distinct recommendations for each group.

Group A: Non-concurrent platinum-based strategies

In this population, the evidence is dominated by small phase I-II experiences and a single phase III trial, with marked heterogeneity in both efficacy and safety. The contrast between the phase II series is instructive: Zhang et al. [19], without any systemic therapy, reported a median PFS of 40 months with no grade ≥3 toxicity, whereas Parisi et al. [18], combining accelerated radiotherapy with neoadjuvant and consolidative chemotherapy, reported a median PFS of only 9.7 months and 70% grade 4 chemo-related toxicity.

So, in frail patients, the toxicity burden is driven largely by the chemotherapy component rather than by HypoRT itself, and a well-conformed high-BED radiotherapy-only schedule can be both effective and remarkably well tolerated. On the other hand, the pembrolizumab-based SPRINT approach [17] (induction immunotherapy, PET-adapted dose, consolidation immunotherapy; mPFS 26 months, 28% grade 3 and no grade 4–5 events) illustrates a biologically rational, chemotherapy-sparing alternative that deserves prospective confirmation.

The only randomized phase III trial in poor-performance-status patients, presented by Iyengar et al. [10], compared 60 Gy in 30 fractions, 2 Gy/fr with an accelerated hypofractionated arm, 60 Gy in 15 Gy, and 4 Gy/fraction, and found no significant overall survival difference but numerically superior local control favoring hypofractionation (85.8% vs. 66.1%; *p* = 0.34). The same trial reported significantly more grade 2 toxicity in the hypofractionated arm, 47 events in 26 of 50 patients (52.0%) against 17 events in 11 of 46 (23.9%), *p* = 0.006, and a numerically shorter median overall survival with hypofractionation, 8.2 against 10.6 months. Radiation-attributable grade 3–5 events occurred in 18 of 50 and 19 of 46 patients, respectively, so the local-control advantage was not obtained at the cost of more severe toxicity, but neither was it accompanied by a survival gain.

Taken together, Group A data suggest not administering peri-radiotherapy chemotherapy with hypofractionated RT, but rather suggest considering dose-escalation strategies guided by metabolic imaging for treatment design and exploring a potential synergistic effect with immunotherapy.

Group B: Concurrent platinum-based strategies

Here, hypofractionation is used not to spare frail patients but to intensify local treatment and compress OTT. Across the nine concurrent studies, the most frequent regimen was platinum-based (most frequently carboplatin-vinorelbine); the median OTT was 4.6 weeks and the median BED10 reached 82 Gy. However, in the concurrent setting, uncontrolled escalation of dose and intensity may reduce the advantage of local-control through fatal normal-tissue toxicity: the median study-level grade 5 toxicity was 5.5% (range 0–17.8%), with 34 fatal events among 431 evaluable patients (7.9%), and reached 20.8% (11/53) in the PET-subvolume arm of the PET-Boost trial and 22.2% (2/9) in the highest dose-escalation cohort of Wu et al., the highest single arm-level figure being 33.3% (2/6) in arm 3 of CALGB 31102 (Supplementary Table S2).

The studies by Wu et al. [22], Cooke [25] and Qiu et al. [23] used selective dose-escalation strategies, increasing the dose to residual disease after induction with chemotherapy or radiotherapy based on metabolic imaging. However, although they reported one-year local control rates ranging from 80% to 97%, arm-level grade 5 toxicity rates of up to 20.8% (11/53) make this approach not advisable in routine clinical practice; it is therefore particularly recommended that this is carried out within the framework of a clinical trial.

### 4.1. Systemic Therapy and the Contemporary Landscape

Two recent developments must temper the interpretation of older studies in this review. The negative PACIFIC-2 trial [38] showed that adding durvalumab concurrently with chemoradiotherapy did not improve PFS or OS (PFS HR 0.85, *p* = 0.247; OS HR 1.03) and increased early toxicity, reinforcing that immunotherapy belongs in the consolidation rather than the concurrent phase. In EGFR-mutated unresectable stage III disease, the LAURA trial established osimertinib after definitive chemoradiotherapy as a new standard, with a median PFS of 39.1 versus 5.6 months (HR 0.16) [39]. These trials underline that any future hypofractionation strategy will have to be designed around—not instead of—molecularly stratified consolidation therapy, and that the lymphocyte-sparing rationale for shortening OTT is most compelling precisely when effective consolidation immunotherapy or targeted therapy is to follow.

Moreover, the integration of hypofractionation with contemporary systemic therapy remains conceptual because the evidence is scarce. Of the 644 patients in the thirteen photon studies, one received consolidation durvalumab; the last participant enrolled by Wu et al. developed only grade 2 acute esophagitis and no late pulmonary toxicity; Katsuta et al. state that no patient received adjuvant durvalumab. Only Ohri et al. integrated a checkpoint inhibitor by design, giving three cycles of induction pembrolizumab, then risk-adapted radiotherapy without chemotherapy, then further pembrolizumab, in patients selected by PD-L1 TPS ≥ 50%. Two implications follow. First, shortening radiotherapy from six weeks to three advances the consolidation window by approximately three weeks: PACIFIC randomized patients 1 to 42 days after radiotherapy, and its post hoc comparison of under versus at least 14 days gave progression-free survival hazard ratios of 0.39 and 0.63 [40], although this timing advantage has not been reproduced in real-world cohorts. Second, no included study stratified on molecular status and only Katsuta et al. report it, with EGFR mutations in 5 of 36 patients. Since the LAURA trial established osimertinib after chemoradiotherapy in EGFR-mutated unresectable stage III disease (median progression-free survival 39.1 versus 5.6 months, hazard ratio 0.16), a hypofractionated course preceding years of a tyrosine kinase inhibitor that carries its own risk of interstitial lung disease is untested. Therefore, it is advisable that PD-L1 and molecular status be recorded, in future hypofractionation trials.

### 4.2. Clinical and Technical Questions

A recurring theme across both groups is that the quality of the radiotherapy—not merely the prescription—determines the balance between local control and toxicity. Only 4 out of 7 Group A studies and 9 out of 14 Group B studies used 4DCT or slow-CT simulation, today’s expected minimum for thoracic motion management. The trials with the most favorable therapeutic ratios consistently combined 4DCT-based simulation, PET/CT for target delineation, intensity-modulated delivery, image guidance and explicit OAR constraints; conversely, the fatal toxicities clustered in the most aggressively escalated cohorts. A subgroup analysis from the Wu trial is illustrative: patients with a smaller boost ITV (bITV) had better PFS than those with a larger bITV, underscoring that the volume of high-dose tissue—not just the prescribed dose—governs the therapeutic ratio. These observations support defining minimum technical standards—4DCT simulation, PET/CT-based GTV definition, ITV-based motion management, IMRT/VMAT delivery, daily IGRT and prospectively specified OAR constraints—as eligibility criteria for any future prospective hypofractionation trial in this setting.

These standards are not of uniform evidential strength, and Table 11 states the strength of each one explicitly. Each recommendation is graded against the consensus vocabulary already used by the field—the Mandatory, Recommended, Optional and Discouraged categories of the ESTRO ACROP target-volume guideline [41]—and against the current dosimetric consensus [42,43] for a 60 Gy in 15 fraction schedule, distinguishing constraints for which those documents cite outcome data from those they label panel consensus. Because the thirteen included studies were all published before either dosimetric document appeared, the comparison establishes the reference standard for the recommendations made here and is not a retrospective audit of the conduct of the included trials.

One multi-arm trial addresses this question directly. NRG-LU004 (ARCHON-1; NCT03801902) is a phase I study in PD-L1-high locally advanced NSCLC in which two initial arms, now closed to accrual, compared accelerated hypofractionated radiotherapy (60 Gy in 15 fractions) with conventional fractionation (60 Gy in 30 fractions), both combined with durvalumab initiated two weeks before radiotherapy; the preliminary safety analysis showed a favorable toxicity profile [44]. The trial continues with two further arms that add either monalizumab (an anti-NKG2A monoclonal antibody) or oleclumab (an anti-CD73 monoclonal antibody) to durvalumab plus conventionally fractionated radiotherapy (60 Gy in 30 fractions).

Moreover, SWOG S1933 (NCT04310020), a pilot study of hypofractionated radiotherapy followed by atezolizumab consolidation in patients with stage II or III NSCLC and borderline performance status, completed accrual in February 2026, and we are currently awaiting the results.

## 5. Limitations

The evidence body is dominated by small, single-arm, heterogeneous studies spanning more than a decade of evolving staging, imaging and delivery technology; only one randomized trial directly compared hypofractionation with conventional fractionation, and it was conducted in poor-performance-status patients. BED comparisons assume an α/β of 10 and do not capture the differential late-toxicity risk of large fractions to central and ultracentral structures. Reporting of lymphopenia, immunotherapy consolidation and molecular subtypes was inconsistent, limiting direct integration with the contemporary PACIFIC/LAURA paradigm. Moreover, the included evidence predates the current standard of care. Only one of the 644 patients in the thirteen photon studies received consolidation durvalumab and only one trial integrated a checkpoint inhibitor by design. In this sense, our recommendations are therefore made on radiotherapy endpoints alone and require prospective confirmation in populations in whom consolidation systemic therapy is planned. Finally, restricting inclusion to English-language, fully published studies may have introduced selection bias. These limitations reinforce, rather than contradict, the central message: radical-intent hypofractionation in unresectable NSCLC is promising but still investigational, and its responsible use depends on careful patient selection and rigorous technical execution.

## 6. Conclusions

Radical-intent hypofractionated radiotherapy is a feasible strategy across the spectrum of unresectable stage II–III NSCLC, but the current evidence is scarce, heterogeneous and largely non-randomized, so its application in daily clinical practice should be approached with caution, preferably within a clinical trial framework. However, two scenarios are clearly distinct: Where concurrent chemotherapy is not being delivered, hypofractionated radiotherapy alone or combined with immunotherapy offers a high-BED radical option with an acceptable toxicity profile. The only randomised support for this in a population explicitly selected for unfitness comes from Iyengar et al., who enrolled patients with Zubrod performance status 2 or worse, weight loss, or physician-assessed ineligibility for chemotherapy, and who reported 2-year local relapse-free survival of 85.8% with hypofractionation versus 66.1% with conventional fractionation without a survival difference. The remaining non-concurrent studies enrolled patients with performance status 0–1 and in most cases delivered sequential systemic therapy, so they support the use of a non-concurrent hypofractionated strategy but do not extend the evidence to frail populations.

In contrast, in candidates for concurrent-based strategies, concurrent hypofractionation can shorten overall treatment time and intensify local therapy, but uncontrolled dose escalation carries a clinically significant risk of fatal toxicity, so the balance must be considered carefully. In this subgroup, selective PET-guided dose escalation with strict normal-tissue sparing should be mandatory. Across both scenarios, modern techniques (4DCT simulation, PET/CT-based target delineation, IMRT/VMAT, daily IGRT and explicit OAR constraints) are the principal determinants of a favorable therapeutic ratio. On the evidence assembled here, radical-intent hypofractionation should target a BED10 of 80–100 Gy, corresponding to an EQD2 of 67–83 Gy, to the highest-dose volume, delivered in 10 to 25 fractions with an overall treatment time not exceeding four weeks. Escalation beyond BED10 100 Gy should be restricted to metabolically defined subvolumes and, outside a clinical trial, should not be combined with concurrent platinum.

Future prospective trials should harmonize these minimum technical standards, prespecify lymphopenia and consolidation systemic therapy, and be designed to complement—rather than replace—the contemporary PACIFIC and LAURA treatment paradigms.

## Figures and Tables

**Table 1:** Population A: no concurrent chemotherapy, stage II–III, photon therapy and 4DCT simulation.

Study	N	Induction	Arm/Vol	DT	DF	Fr	BED10/EQD2	Weeks	Adjuvant Chemo	mFU	mPFS	mOS	LC	Toxicity G ≥ III
Ohri et al. 2024 (SPRINT) Phase II [17]	25	Pembro 200 mg/21 d × 3; PD-L1 ≥ 50%	PET ≤ 20 cc (11 pt)	48	2.4	20	59.52/49.6	4	Pembro 1 y (16 cycles)	22	26	Nr (76% 2 y)	NR	28% 7 p G3; 0% G4; 0% G5
			PET > 20 cc (14 pt)	55	2.75	20	70.13/58.44							
Parisi et al. 2019 Phase II [18]	17	CDDP 75/Tax 75 × 2	pPTV	30	6	5	48/40	1	CDDP 75/Tax 60 × 2	87	9.7	23	77%	Chemo-related: 70% G4; RT-related: 0%
			nPTV	25	5	5	37.5/31.25							
Zhang W et al. 2015 Phase II [19]	28	No (SIB)	iGTV	75	5	15	112.5/93.7	3	No	41	40	46.5	74% 3 y	0%
			CTV	60	4	15	84/70							
			PTV	45	3	15	58.5/48.75							
Iyengar P 2021 Phase III [10]	96	Chemo 7p	N:46 (conv)	60	2	30	72/60	6	Adjuvant chemo (30p)	8.7	7.3	10.6	66.1% 2 y	41.3%,19 p (G ≥ 3)
			N:50 (hypo)	60	4	15	84/70	3			6.4	8.2	85.8% 2 y	36%, 18 p (G ≥ 3)

Abbreviations: DT, total dose (Gy); DF, dose per fraction (Gy); Fr, fractions; N, patients; BED, biologically effective dose (Gy); EQD2, equivalent dose in 2 Gy/fr (α/β = 10); mFU, median follow-up (months); mPFS, median progression-free survival (months); mOS, median overall survival (months); LC, local control; NR, not reported; Nr, not reached; SIB, simultaneous integrated boost; iGTV, internal gross tumor volume; CTV, clinical target volume; PTV, planning target volume; pPTV, primary-tumor PTV; nPTV, nodal PTV; p, patients.

**Table 2:** Population B: Phase I, stage II–III, photon therapy, concurrent chemotherapy, 4DCT simulation.

Study	N	Arms	DT	DF	Fr	N	BED10/EQD2	Weeks	Chemo	mFU	mPFS	mOS	LC	Toxicity ≥ GIII
Kim et al. 2017 [20]	12	48 Gy/2.4 Gy/20 fx +Boost	Boost:16.8	2.8	7	5	89.99/74.9	5.4	CDDP + VP16/28 d	22	NR	21.7	81% (1 y)	0p
		48 Gy/2.4 Gy/20 fx +Boost	Boost:20	2.9	7	3	95.29/79.42	5.4						0p
		48 Gy/2.4 Gy/20 fx +Boost	Boost:22.7	3.2	7	4	101.94/84.96	5.4						25%, 1p G3
Urbanic JJ et al. 2018 [21]	21	Arm 1	60	2.2	27	6	72.47/60.39	5.4	* CBDCA/w + Paclitaxel	NR				16%, 1p G3
		Arm 2	60	2.5	24	6	75/62.5	4.8	CBDCA/w + Paclitaxel					16%, 1p G3; 16% 1p G5
		Arm 3	60	2.72	22	6	76.38/63.65	4.4	CBDCA/w + Paclitaxel					33%, 2p G5
		Arm 4 **	60	3	20	1	78/65	4.0	CBDCA/w + Paclitaxel					0
		Total	60			19					12.2	19.3	76%	5p 26%
Wu et al. 2024 [22]	28	Sequential boost (low)	40+25	5	5	10	93.5/77.9	2	CBDCA AUC2 + Paclitaxel 45 mg/m^2^/w	14.3	9.8	15.3	74.1% (2 y)	20%, 2p G3
		(intermediate)	40+30	6	5	9	104/86.67	2		41.7	11.2	42.5	85.7% (2 y)	0%
		(high)	40+35	7	5	9	115.5/96.25	2		24.2	10.5	Nr	100% (2 y)	22%; 2p G5

Abbreviations as in Table 1. CDDP, cisplatin; VP16, etoposide; CBDCA, carboplatin; AUC, area under the curve; w, weekly; DT, Total Dose. * Concurrently and 2 cycles of consolidation; ** Not completed due to dose limiting toxicity; NR: not reported.

**Table 3:** Population B: Phase II, stage II–III, photon therapy, concurrent chemotherapy, 4DCT simulation.

Study		N	Induction	DT	DF	Fr	N	BED10/EQD2	Weeks	Chemo	mFU	mPFS	mOS	LC	Toxicity ≥ GIII
Qiu B et al. 2021 (GASTO1011) [23]		99	51 Gy/3 Gy/17 fr → if no prog/tox boost	15–18	3	5–6	89	84.15/70.13	5	Docetaxel 25 + Nedaplatin 25 mg/m^2^	29.5	11	27	66.5% (2 y)	Acute esophagitis 15/89 (16.9%); acute pneumonitis 7/89 (7.9%); late pneumonitis 2/89 (2.2%). 3.3% G5 (3/89 boosted; 3/99 enrolled)
Van Diessen et al. 2020 [24]		47	—	66	2.75	24	47	82.3/68.58	5	CDDP 6 mg/m^2^/d ± Cetuximab	67	NR	33	NR	NR
Cooke et al. 2023 [25]	Arm A: Whole tumor	107	—	79	3.30	24	54	105.34/87.78	5	CDDP (Stage III)	73	46% (1 y)	18	97% (1 y)	65% 35/54 G3; 14.8% 8/54 G5
	Arm B: PET subvolume		—	84	3.50	24	53	113.4/95.50	5			43% (1 y)	18	91% (1 y)	70% 37/53 G3; 20.8% 11/53 G5
Roy et al. 2016 [26]		36	CBDCA AUC5 + Paclitaxel 200 mg/m^2^ × 2	60	2	30	18	72/60	6	—	15	5.36	12.33	NR	8p G3; 1p G5
				48	2.4	20	18	59.52/49.6	4	CDDP 30 mg/m^2^/w		17	24.73	NR	4p G3; 1p G5
Glinski et al. 2020 [27]		92	—	58.8	2.8	21	92	75.26/62.7	4	CDDP 80 mg/m^2^ + VNR	21.5	25	38	Nr	14.1% 13/92 G3; 5.4% 5/92 G5
Katsuta et al. 2021 [28]		36	—	67.5	2.5	27	36	84.3/70.3	6	CDDP + VNR	50	10.7	NR	79.8% (1 y); 66% (3 y)	14% (5p) G3

Abbreviations as in Table 1 and Table 2. Cooke et al. also included stage IB; VNR, vinorelbine; the PET subvolume included the region representing 50% of the initial SUVmax. Early closed for slow accrual.

**Table 4:** BED Ranges.

Stratum	BED10-High	EQD2	Studies (A/B)	Pts	mPFS (mo)	Local Control	G ≥ 3	G 5
**I—Low**	<60 Gy	<50 Gy	Ohri (PET ≤ 20 cc), Parisi/Roy (hypofractionated arm)	46	9.7–26	77%	0–70%	1/46 (2.2%)
**II—Standard**	60–79.9 Gy	50–66.6 Gy	Ohri (PET > 20 cc), Iyengar (conventional arm)/Urbanic, Roy (conventional arm), Glinski	191	5.4–26	66.1–76%	14.1–41.3%	9/145 evaluable (6.2%)
**III—Escalated**	80–99.9 Gy	66.7–83.2 Gy	Iyengar (hypofractionated arm)/Kim (levels 1–2), Wu (low), Qiu, Van Diessen, Katsuta	250	6.4–11	66.5–85.8% at 2 y	0–36%	3/153 evaluable (2.0%)
**IV—Ablative**	≥100 Gy	≥83.3 Gy	Zhang/Kim (level 3), Wu (intermediate, high), Cooke A–B	157	10.5–NR	85.7–100% at 2 y	0–70%	21/157 (13.4%)

**Table 5:** NIH/NHLBI quality assessment of single-arm studies (Case Series tool).

Study	1	2	3	4	5	6	7	8	9	Rating
Ohri 2024 et al. (SPRINT) [17]	**+**	**+**	**+**	**+**	**+**	**+**	**+**	**+**	**+**	**Good**
Parisi et al. 2019 [18]	**+**	**+**	**CD**	**+**	**+**	**+**	**+**	**+**	**+**	**Good**
Zhang W et al. 2015 [19]	**+**	**+**	**CD**	**+**	**+**	**+**	**+**	**+**	**+**	**Good**
Kim et al. 2017 [20]	**+**	**+**	**CD**	**+**	**+**	**+**	**+**	**+**	**+**	**Fair**
Qiu 2021 et al. (GASTO1011) [23]	**+**	**+**	**+**	**+**	**+**	**+**	**+**	**+**	**+**	**Good**
Wu 2024 et al. [22]	**+**	**+**	**+**	**+**	**+**	**+**	**+**	**+**	**+**	**Good**
Hoppe 2022 et al. [13]	**+**	**+**	**CD**	**+**	**+**	**+**	**+**	**+**	**+**	**Fair**
Contreras et al. 2022 [14]	**+**	**+**	**CD**	**+**	**+**	**+**	**+**	**+**	**+**	**Fair**
Gómez et al. 2013 [15]	**+**	**+**	**CD**	**+**	**+**	**+**	**+**	**+**	**+**	**Fair**
Takahashi et al. 2015 [11]	**+**	**+**	**CD**	**+**	**+**	**+**	**CD**	**+**	**+**	**Fair**
Saitoh et al. 2018 [12]	**+**	**+**	**CD**	**+**	**+**	**+**	**+**	**+**	**+**	**Fair**
Liu et al. 2013 [29]	**+**	**+**	**CD**	**+**	**+**	**+**	**CD**	**+**	**+**	**Fair**
Aye et al. 2021 [30]	**+**	**+**	**CD**	**+**	**+**	**+**	**CD**	**+**	**+**	**Fair**
Westover et al. 2015 [5]	**+**	**+**	**CD**	**+**	**+**	**+**	**CD**	**+**	**+**	**Fair**
Cannon et al. 2013 [7]	**+**	**+**	**CD**	**+**	**+**	**CD**	**CD**	**CD**	**+**	**Fair**
Katsuta et al. 2021 [28]	**+**	**+**	**CD**	**+**	**+**	**+**	**+**	**+**	**+**	**Fair**
Ren et al. 2016 [31]	**+**	**+**	**+**	**+**	**+**	**+**	**−**	**+**	**+**	**Fair**
Glinski et al. 2020 [27]	**+**	**+**	**CD**	**+**	**+**	**+**	**+**	**+**	**+**	**Fair**
Zhu et al. 2011 [32]	**+**	**+**	**CD**	**+**	**+**	**CD**	**+**	**CD**	**+**	**Fair**
Lin et al. 2013 [33]	**+**	**+**	**CD**	**+**	**+**	**+**	**+**	**+**	**+**	**Fair**
Agolli et al. 2015 [8]	**+**	**+**	**CD**	**+**	**+**	**CD**	**+**	**+**	**+**	**Fair**
Van Diessen 2020 [24]	**+**	**+**	**+**	**+**	**+**	**+**	**+**	**+**	**+**	**Good**
Cagney et al. 2018 [34]	**+**	**+**	**CD**	**+**	**+**	**+**	**+**	**+**	**+**	**Fair**
Walraven et al. 2016 [35]	**+**	**+**	**CD**	**+**	**+**	**+**	**+**	**+**	**+**	**Fair**
Urbanic et al. 2018 [21]	**+**	**+**	**+**	**+**	**+**	**+**	**+**	**+**	**+**	**Good**
Li et al. 2020 [36]	**+**	**+**	**CD**	**+**	**+**	**+**	**+**	**+**	**+**	**Fair**
Zygogianni et al. 2020 [9]	**+**	**+**	**CD**	**+**	**+**	**CD**	**+**	**+**	**+**	**Fair**
de Haan 2021 et al. [6]	**+**	**+**	**CD**	**+**	**+**	**+**	**+**	**+**	**+**	**Fair**

Items: 1, objective clearly stated; 2, study population/case definition; 3, consecutive cases; 4, comparable subjects; 5, intervention clearly described; 6, outcomes valid/reliable/consistent; 7, adequate follow-up; 8, statistical methods described; 9, results well described. Ratings: + met; − not met; CD cannot determine/NR not reported.

**Table 6:** NIH/NHLBI quality assessment of randomized trials (Controlled Intervention tool).

Study	1	2	3	4	5	6	7	8	9	10	11	12	13	14	Rating
Iyengar et al. 2021 (Ph III) [10]	**+**	**+**	**CD**	**−**	**CD**	**+**	**+**	**NA**	**+**	**+**	**+**	**CD**	**+**	**+**	**Fair**
Roy 2016 et al. (Ph II rand.) [26]	**+**	**CD**	**CD**	**−**	**CD**	**+**	**+**	**NA**	**+**	**+**	**+**	**CD**	**+**	**+**	**Fair**
Cooke 2023 et al. (PET-Boost) [25]	**+**	**+**	**CD**	**−**	**CD**	**+**	**+**	**NA**	**+**	**+**	**+**	**+**	**+**	**+**	**Fair**

Items: 1, described as randomized; 2, adequate randomization method; 3, allocation concealment; 4, participants/providers blinded; 5, outcome assessors blinded; 6, baseline comparability; 7, drop-out ≤ 20%; 8, differential drop-out ≤ 15 pp; 9, high adherence; 10, other interventions similar/avoided; 11, valid/consistent outcome measures; 12, adequate power; 13, prespecified outcomes; 14, intention-to-treat analysis. Ratings: + met; − not met; CD cannot determine; NA not applicable.

**Table 7:** GTV imaging modality and CTV/PTV margins by study.

Study	GTV Imaging	CTV (Primary)	CTV (Nodal)	PTV Margin
Takahashi et al. 2015 [11]	NR (carbon)	GTV + 10 mm + involved/uninvolved regional nodes	Included (ENI)	CTV + 5 mm
Saitoh et al. 2018 [12]	CT (carbon)	GTV + prophylactic hilar/mediastinal nodes	ENI	PTV1 = CTV + 5–10 mm; PTV2 = GTV + 5–10 mm; PTV3 near OAR
Liu et al. 2013 [29]	CT	GTV + 8 mm (adeno)/6 mm (other)	NR	CTV + 10–15 mm (respiratory motion)
Kim et al. 2017 [20]	PET (MIP)	ITV-based	ITV-based	ITV + 10 mm
Hoppe et al. 2022 [13]	End-inhalation CT (proton)	CTV-HD = iGTV + 6 mm	CTV-LD = CTV-HD + adjacent station	PTV = CTV + 5 mm
Contreras et al. 2022 [14]	PET (proton)	iGTV-P + 5 mm	iGTV-N	CTV + 5 mm
Aye et al. 2021 [30]	CT	GTV + 8 mm	GTV + 8 mm	CTV + 10 mm
Ohri 2024 et al. (SPRINT) [17]	PET	iGTV + 7–10 mm	iGTV + 7–10 mm	CTV + 5 mm
Kenneth D Westover et al. 2015 [5]	CT	GTV-P + 5–10 mm	GTV-N + 5–10 mm	CTV + 5–10 mm
Parisi et al. 2019 [18]	PET	NA (no ENI)	NA	NA
Gómez et al. 2013 [15]	PET or CT (proton)	iGTV + 5 mm	—	iGTV + 5 mm
Cannon et al. 2013 [7]	PET and/or CT	iGTV + 6 mm	iGTV + 6 mm	CTV + 2 mm
Wu et al. 2024 [22]	PET	iGTV + 5 mm; boost: residual iGTV, no margin	—	iGTV + 5 mm; boost: no margin
Katsuta et al. 2021 [28]	PET	GTV-P + 3–5 mm	GTV-N=CTV-N (esophagus sparing)	CTV + 5–15 mm
Ren et al. 2016 [31]	CT	Reported in companion publication	—	NR
Glinski et al. 2020 [27]	PET	3D: GTV + 5 mm; 4D: iGTV + 5 mm	same	3D: CTV + 10 mm; 4D: CTV + 5 mm
Zhu et al. 2011 [32]	CT ± PET	NR	NR	GTV + margin (unspecified)
Lin et al. 2013 [33]	CT and PET	GTV-N + 6–8 mm	NR	CTV-P + 6–10 mm (respiratory motion)
Agolli et al. 2015 [8]	CT and PET	GTV-P + 5 mm	GTV-N + 5 mm	CTV + 5 mm
Van Diessen et al. 2020 [24]	PET and CT	NR	NR	NR
Zhang W et al. 2015 [19]	CT ± PET	GTV-P + 5–7 mm	NR	CTV-P + 5 mm
Iyengar et al. 2021 [10]	CT (>1 cm) ± PET (SUV > 3)	NR	NR	NR
Cagney et al. 2018 [34]	CT ± PET	GTV-P + 5 mm	NR	CTV-P + 10–15 mm
Walraven et al. 2016 [35]	PET and CT	NR	NR	GTV-P + 12 mm
Urbanic et al. 2018 [21]	CT ± PET	NR	NR	NR
Qiu et al. 2021 [23]	CT ± PET	GTV-P	NR	GTV-P + 5–7 mm
Li et al. 2020 [36]	PET and CT (>15 mm)	GTV-P + 5–8 mm	GTV-N + 5 mm	CTV + 5 mm
Zygogianni et al. 2020 [9]	CT ± PET	GTV-P + 5–8 mm	GTV-N + 5 mm	CTV + 5 mm
de Haan et al. 2021 [6]	PET and CT	NR	NR	GTV-P + 12 mm + ¼ motion; GTV-N + 9–12 mm
Cooke 2023 et al. (PET-Boost) [25]	PET or CT	GTV-P + 5 mm (center-dependent)	GTV-N + 5 mm (optional)	Individualized per center
Roy 2016 et al. [26]	CT and PET	No ENI	NR	CTV (primary + nodal) + 10–15 mm

Abbreviations: GTV, gross tumor volume; CTV, clinical target volume; PTV, planning target volume; iGTV, internal GTV; ITV, internal target volume; ENI, elective nodal irradiation; HD, high dose; LD, low dose; MIP, maximum-intensity projection; NR, not reported; NA, not applicable.

**Table 8:** Most frequently used organ-at-risk constraints across the analyzed studies.

Organ at Risk	Most Frequently Used Metric	Typical Reported Constraint(s)
Lungs	V20 and/or mean lung dose (MLD)	V20 ≤ 30–35%; MLD ≤ 16–20 Gy (most common pair)
Spinal cord	Maximum dose (Dmax)	Dmax ≤ 40–50 Gy (typically ≤ 45 Gy)
Esophagus	Mean dose, Dmax, V35-V55, irradiated length	Mean ≤ 34 Gy; Dmax ≤ 45–70 Gy; V35 ≤ 65%; or length-based
Heart	V40 and/or mean dose	V40 ≤ 40–50%; mean ≤ 20 Gy
Brachial plexus	Maximum dose (Dmax)	Dmax ≤ 52–79 Gy (reported only in particle/ultracentral series)
Mainstem bronchus	Maximum dose/small-volume dose	Dmax ≤ 60–84 Gy (reported in a minority of studies)

Abbreviations: V20, volume receiving ≥ 20 Gy (%); MLD, mean lung dose; Dmax, maximum dose.

**Table 9:** Eligibility Criteria of the Included Studies.

Study	Group	Performance Status Required	Age Limit	Pulmonary Function Threshold
Ohri 2024 (SPRINT) [17]	A	ECOG 0–1	None stated	None stated
Parisi 2019 [18]	A	PS 0–1	<70 years	FEV1 > 50% predicted
Zhang 2015 [19]	A	Karnofsky recorded, median 70 (range 60–90); no threshold stated	Not stated	None stated; planning constraints of bilateral lung V20 < 30% and heart V25 < 50%
Iyengar 2021 [10]	A	Zubrod ≥ 2	None stated	None stated
Kim 2017 [20]	B	ECOG ≤ 1	≥18 years	FEV1 ≥ 1.0 L and DLCO ≥ 40% predicted
Urbanic 2018 (CALGB 31102) [21]	B	ECOG 0–1 (67% ECOG 1)	≥18 years	FEV1 ≥ 1.2 L or ≥50% predicted, plus diffusion capacity
Wu 2024 [22]	B	Karnofsky ≥ 70	≥18 years	None stated
Qiu 2021 (GASTO1011) [23]	B	ECOG 0–1 (83.1% ECOG 0)	18–75 years	FEV1 > 0.8 L, FEV1/FVC% ≥ 40%, DLCO% ≥ 45%
Van Diessen 2020 [24]	B	WHO 0–1	Not stated	Not stated
Cooke 2023 (PET-Boost) [25]	B	ECOG ≤ 2 (ECOG 2 in 5/54 and 2/53)	≥18 years	“Adequate” pulmonary function, not quantified
Roy 2016 [26]	B	ECOG 0–1	Not stated	No significant pulmonary impairment
Glinski 2020 [27]	B	Karnofsky 80–100	<75 years	None stated
Katsuta 2021 [28]	B	ECOG 0–2	<81 years	None stated; total-lung V20 Gy > 35% rendered a patient ineligible

**Table 10:** Systematic Synthesis of Toxicity.

Study	Esophageal G ≥ 3	Pulmonary G ≥ 3	Fatal Hemorrhage	Hematologic G ≥ 3	Basis
Ohri 2024 [17]	Esophagitis 1 (4%); G ≥ 2 = 9 (36%)	Pneumonitis 1 (4%); G ≥ 2 = 2 (8%)	0	Anemia 1 (4%); G ≥ 2 = 2 (8%)	Events; ≤7 patients of 25; no G4 or G5
Zhang 2015 [19]	0 (G1 esophagitis 2/28)	0 (G1–2 pneumonitis 8/28, G1 fibrosis 5/28)	0	Not applicable (no systemic therapy)	Patients, N = 28; toxicity assessed to 3 months only
Parisi 2019 [18]	Not reported separately	Late dyspnea 4/17 (24%); text calls these pneumonitis	0	G4 neutropenia 12, G4 leucopenia 4, G3 leucopenia 5—all chemotherapy-attributed	By term
Iyengar 2021 hypofx [10]	Esophagitis 1, dysphagia 1; G2 esophagitis 11/50 (22.0%)	Dyspnea 8, ARDS 1, cough 1, pleural effusion 1, pneumonitis 1	0	Not reported	Events; G3–5 patient total 18/50
Iyengar 2021 conv [10]	Esophagitis 1, dysphagia 1; G2 esophagitis 4/46 (8.7%)	Dyspnea 3, pneumonia 4, pneumonitis 1, pleural effusion 1	0	Not reported	Events; G3–5 patient total 19/46
Kim 2017 [20]	0	0	0	0	Patients; only G3 in trial showed fatigue during consolidation chemotherapy
Urbanic 2018 [21]	Acute esophagitis excluded from the DLT definition by protocol	Lung infection 1 (G3); pneumonitis 1 (G5)	2/21 (9.5%) at 9 months	Excluded from the DLT definition by protocol	Events
Wu 2024 [22]	Not reported separately	Not reported separately	2/9 (22.2%) in the high-dose cohort	Not reported	—
Qiu 2021 [23]	Esophagitis 15 (16.9%); no G ≥ 4 acute non-hematologic; G2 esophagitis 45 (50.6%)	Acute pneumonitis 7 (7.9%); late pneumonitis G3 = 2 (2.2%); G2 pneumonitis 28 (31.5%)	2/89 (2.2%), both central squamous	Lymphopenia G3 = 50 (56.2%) + G4 = 19 (21.3%) = 69/89 (77.5%); thrombocytopenia G3 = 5, G4 = 1; leukopenia G3 = 5; neutropenia G3 = 3	Events by term
Cooke 2023 arm A [25]	Dysphagia/esophagitis 19%	Pulmonary events 19%	4/54 (7.4%)	Not reported	Authors’ own Discussion figures; G ≥ 3 and 54% of patients
Cooke 2023 arm B [25]	Dysphagia/esophagitis 17%	Pulmonary events 32%	3/53 (5.7%)	Not reported	As above; G ≥ 3 and 53% of patients
Roy 2016 arm A [26]	0	Pneumonitis 1/18 (5.6%)	0	2/18 (11.1%)	Patients
Roy 2016 arm B [26]	Pharyngitis/esophagitis 1/18 (5.6%)	0	0	1/18 (5.6%)	Patients
Glinski 2020 [27]	13/92 (14.1%), of which 1 G4 and 1 fatal; all but one lasted >2 weeks	Early G3 pneumonitis 3/92 (3.3%); late G3 pulmonary 2/92 at 6 months	≥2/92 (2.2%): 1 definite at 2 months after completion, 1 probable at 2 days after completion (pulmonary artery invasion, pretreatment hemoptysis)	G4 neutropenia 17/92 (18.5%); no G3–4 thrombocytopenia or anemia; prolonged G3–4 neutropenia curtailed cycle 2 in 13/92	Patients; early G ≥ 3 non-hematologic 16/92 (17.4%)
Katsuta 2021 [28]	Acute esophagitis 1/36 (2.8%) and late stenosis 1/36 (2.8%) in the same patient; G2 esophagitis 22/36 (61.1%)	Pneumonitis 3/36 (8.3%), of whom 2 had dilated cardiomyopathy; G2 = 8/36 (22.2%); no late G3 pulmonary event reported	0	Not reported	Patients, n = 36, as tabulated by the authors; distinct patients with G ≥ 3 = 4/36 (11.1%)

**Table 11:** Evidence-based recommendations for moderately hypofractionated thoracic radiotherapy.

Recommendation	Evidence Within This Review (Studies Published 2015–2024)	ACROP 2018 [41]	Current Consensus for 60 Gy/15 fr (ASTRO, Online 2025; French Update, 2026 Volume)—Postdates All Included Studies	Grade
Diagnostic FDG-PET/CT before target definition	GTV defined on PET/CT in roughly half of cohorts; no comparison of PET-defined vs. CT-defined outcomes	Mandatory (M); planning PET/CT (R); 4D-PET/CT (O)	Not addressed (dosimetric documents)	C
A respiratory-motion management strategy (ITV/iGTV, mid-ventilation or mid-position, or gating/tracking)	Inclusion criterion, so not testable here; PTV margins clustered at ~5 mm where iGTV was used vs. 10–15 mm where it was not (descriptive, Table 4)	4D-CT assessment (R); one of three motion policies must be chosen; PTV margin for geometric uncertainty (M); manual PTV editing (D)	Constraints defined on lungs–iGTV or lungs–ITV, presupposing motion management	C
Involved-field rather than elective nodal irradiation	Katsuta et al.: elective nodal failure in 3/36 (8.3%)	Elective nodal CTV not recommended (D)	Not addressed	B
Department-specific rather than fixed PTV margin	Association only; no margin was tested against outcome	Residual errors should be quantified per department (R); anisotropic margins (R)	Not addressed	D
Lung: V20 ≤ 30–35% and MLD ≤ 16–20 Gy	Most frequently applied pair across the 13 studies; the only validated reference available to them; no dose–response analysis performed	Lungs mandatory to contour (M); no numeric limit given	Now superseded: ASTRO lungs–iGTV V16.5 Gy ≤ 37%, V5 Gy ≤ 65%, mean ≤ 16.5 Gy (referenced to NRG-LU004); French lungs–ITV mean < 14 Gy, V15 Gy < 20%	Historical record, not a current recommendation; the prospective recommendation is C
Spinal cord/canal Dmax ≤ 45 Gy, tightened to 37–40 Gy for large fractions	Several studies tightened to 37–40 Gy while ≤45 Gy was the accepted limit; no toxicity analysis by cord dose	Spinal canal as PRV mandatory to contour (M)	ASTRO D0.03 cc ≤ 36.5 Gy (primary), ≤40.2 Gy (secondary); French ≤ 39.5 Gy, PRV 3 mm ≤ 42 Gy	C; the tightening studies anticipated the current consensus, which ≤45 Gy exceeds at 4 Gy/fr
Esophagus constraint, structure and metric unresolved	PET-Boost protocol amendment adding an esophagus + 5 mm maximum-dose objective reduced grade 4–5 esophageal toxicity from 5/54 to 0/53, a within-trial comparison independent of any external guideline	Esophagus mandatory to contour, cricoid to gastro-esophageal junction (M)	Discordant: ASTRO D0.03 cc ≤ 58.9 Gy (referenced), mean ≤ 25 Gy (panel consensus); French Dmax < 50.5 Gy, D5 cc < 48 Gy	A for the existence of a maximum-dose objective; the numeric value remains unresolved
Cardiac V40 ≤ 40–50% or a mean-dose limit	Descriptive convention; no cardiac-event analysis	Whole heart mandatory to contour, substructures optional (O)	ASTRO D0.03 cc ≤ 60 Gy (panel consensus), mean ≤ 16.5 Gy (literature); French Dmax < 66 Gy, D10 cc < 62 Gy	D for the maximum-dose element, C for mean dose
Great vessels: avoid escalation with ≥50% encasement; respect a maximum-dose limit	All fatal hemorrhages in PET-Boost had ≥50% or complete encasement at mediastinal PRV Dmax 74.4–76.8 Gy, under guidance that then required no vascular constraint; fatal hemorrhage in ≥15 of the 644 patients of the photon cohort (2.3%, 95% CI 1.4–3.8), reported by five independent studies (Supplementary Table S2)	Great vessels do not need to be defined for conventional or moderately hypofractionated RT	ASTRO D0.03 cc ≤ 60 Gy (panel consensus), ≤66 Gy secondary, referenced to early-stage/ultracentral SBRT trials; French Dmax < 64 Gy (mandatory), D10 cc < 60 Gy	B; this review supplies the stage II–III outcome evidence that current consensus lacks
Proximal bronchial tree and brachial plexus should be contoured and constrained	Reported by a minority of studies only; a reporting gap	PBT and trachea not required; brachial plexus optional (O) in superior sulcus or thoracic-inlet tumors	ASTRO brachial plexus D0.03 cc ≤ 49.8 Gy (literature), trachea/bronchus ≤ 60 Gy (panel consensus); French plexus < 50 Gy, PBT < 66 Gy, D10 cc < 62 Gy	C for brachial plexus, D for proximal bronchial tree
Confine ablative dose to a residual metabolic subvolume; the high-dose volume governs the therapeutic ratio	Wu et al.: smaller boost ITV associated with better PFS; Zhang 0/28 grade ≥ 3 at BED10 112.5 Gy with SIB to iGTV vs. Cooke 14.8–20.8% grade 5 at BED10 105–113 Gy with concurrent platinum	Boost volumes not addressed	Not addressed; ASTRO excludes SIB from its scope	B, confounded by concurrent chemotherapy
Selective PET-guided escalation should be confined to trials, not mandated	Arm-level grade 5 toxicity up to 20.8% (11/53) in the most escalated cohorts	Not addressed	Not addressed	B
60 Gy in 15 fractions in patients unfit for concurrent chemotherapy	Iyengar et al., the only randomised comparison: no OS difference, local control 85.8% vs. 66.1% (*p* = 0.34)	Not addressed	This is the schedule for which both current documents provide constraints	A, but underpowered; the local-control difference is not significant

Abbreviation: Grades: A, supported by randomised or within-trial comparative evidence in this setting; B, consistent non-randomised observation across studies included in this review; C, established in international consensus guidelines for this or an adjacent fractionation but not tested within this evidence base; D, expert opinion or convention without supporting outcome data, including items that the source guidelines themselves designate panel consensus.

## Data Availability

The original contributions presented in this study are included in the article. Further inquiries can be directed to the corresponding author.

## Supplementary Materials

The following supporting information can be downloaded at: https://www.mdpi.com/article/10.3390/cancers18182997/s1, Table S1. PRISMA 2020 Checklist [45]; Table S2. Study-level and arm-level audit of fatal (grade 5) toxicity in the 13 photon studies with contemporary simulation.
