# Supplementary material for: Hypofractionation in Unresectable Stage II–III NSCLC: A Systematic Technical Review and Practical Recommendations
Source: Cancers (Basel). 2026 Sep 16;18(18):2997. doi: 10.3390/cancers18182997 (PMC13605718; doi:10.3390/cancers18182997)
Supplement: Supplementary file 1 [file cancers-18-02997-s001.zip › cancers-4472331-supplementary.pdf]

PRISMA 2020 Checklist

Table S1. PRISMA 2020 checklist.

Section and Topic	Item #	Checklist item	Location where item is reported
**TITLE**			
Title	1	Identify the report as a systematic review.	Title (page 1); Abstract, Methods
**ABSTRACT**			
Abstract	2	See the PRISMA 2020 for Abstracts checklist.	Abstract (page 1); Simple Summary
**INTRODUCTION**			
Rationale	3	Describe the rationale for the review in the context of existing knowledge.	Introduction (paragraphs 1-4)
Objectives	4	Provide an explicit statement of the objective(s) or question(s) the review addresses.	Introduction (last paragraph); Abstract, Objective
**METHODS**			
Eligibility criteria	5	Specify the inclusion and exclusion criteria for the review and how studies were grouped for the syntheses.	Material and Methods (paragraphs 2-3, inclusion/exclusion criteria and study grouping into non-photon/photon and non-concurrent/concurrent)
Information sources	6	Specify all databases, registers, websites, organisations, reference lists and other sources searched or consulted to identify studies. Specify the date when each source was last searched or consulted.	Material and Methods (paragraph 1): PubMed, EMBASE, CENTRAL and Web of Science, searched Jan 2010-Jan 2024
Search strategy	7	Present the full search strategies for all databases, registers and websites, including any filters and limits used.	Material and Methods (paragraph 1) and Supplementary data (full search strategy)
Selection process	8	Specify the methods used to decide whether a study met the inclusion criteria of the review, including how many reviewers screened each record and each report retrieved, whether they worked independently, and if applicable, details of automation tools used in the process.	Material and Methods (paragraph 3): six independent reviewers, two-round screening with consensus
Data collection process	9	Specify the methods used to collect data from reports, including how many reviewers collected data from each report, whether they worked independently, any processes for obtaining or confirming data from study investigators, and if applicable, details of automation tools used in the process.	Material and Methods (paragraphs 4-5): manual review and data extraction of

PRISMA

PRISMA 2020 Checklist

Section and Topic	Item #	Checklist item	Location where item is reported
			clinical and technical variables
Data items	10a	List and define all outcomes for which data were sought. Specify whether all results that were compatible with each outcome domain in each study were sought (e.g. for all measures, time points, analyses), and if not, the methods used to decide which results to collect.	Material and Methods (paragraph 5, clinical outcome variables); Results; Tables 1-3
	10b	List and define all other variables for which data were sought (e.g. participant and intervention characteristics, funding sources). Describe any assumptions made about any missing or unclear information.	Material and Methods (paragraph 5, technical variables); Tables 4-5
Study risk of bias assessment	11	Specify the methods used to assess risk of bias in the included studies, including details of the tool(s) used, how many reviewers assessed each study and whether they worked independently, and if applicable, details of automation tools used in the process.	Material and Methods (last paragraph): NIH/NHLBI quality assessment tools; Quality of the Studies analyzed; Tables 6-7
Effect measures	12	Specify for each outcome the effect measure(s) (e.g. risk ratio, mean difference) used in the synthesis or presentation of results.	Material and Methods (paragraph 5); Results (dose, BED10, EQD2, mPFS, mOS, LC, toxicity); Tables 1-3
Synthesis methods	13a	Describe the processes used to decide which studies were eligible for each synthesis (e.g. tabulating the study intervention characteristics and comparing against the planned groups for each synthesis (item #5)).	Material and Methods (paragraphs 2-4); Results (grouping into Group A and Group B)
	13b	Describe any methods required to prepare the data for presentation or synthesis, such as handling of missing summary statistics, or data conversions.	Material and Methods (paragraph 5, BED10/EQD2 calculation with alpha/beta=10); Tables 1-3
	13c	Describe any methods used to tabulate or visually display results of individual studies and syntheses.	Material and Methods; Results; Tables 1-7 (tabular synthesis)
	13d	Describe any methods used to synthesize results and provide a rationale for the choice(s). If meta-analysis was performed, describe the model(s), method(s) to identify the presence and extent of statistical heterogeneity, and software package(s) used.	Not applicable - narrative/qualitative synthesis; no meta-analysis was performed (Results)
	13e	Describe any methods used to explore possible causes of heterogeneity among study results (e.g. subgroup analysis, meta-regression).	Results (Group A vs Group B subgroups); Discussion (heterogeneity by

Section and Topic	Item #	Checklist item	Location where item is reported
			patient fitness and technique)
	13f	Describe any sensitivity analyses conducted to assess robustness of the synthesized results.	Not applicable - no meta-analysis or sensitivity analysis performed
Reporting bias assessment	14	Describe any methods used to assess risk of bias due to missing results in a synthesis (arising from reporting biases).	Not applicable - no quantitative synthesis; reporting bias not formally assessed
Certainty assessment	15	Describe any methods used to assess certainty (or confidence) in the body of evidence for an outcome.	Material and Methods (last paragraph): NIH/NHLBI study-quality assessment; Quality of the Studies analyzed
**RESULTS**			
Study selection	16a	Describe the results of the search and selection process, from the number of records identified in the search to the number of studies included in the review, ideally using a flow diagram.	Results (paragraph 1: 340 records, 170 duplicates, 26/13 studies included); PRISMA flow diagram
	16b	Cite studies that might appear to meet the inclusion criteria, but which were excluded, and explain why they were excluded.	Results (paragraphs 1-2): studies including stage IV excluded [3-7]; non-photon/proton studies excluded from photon analysis
Study characteristics	17	Cite each included study and present its characteristics.	Results; Tables 1-4 (characteristics of each included study)
Risk of bias in studies	18	Present assessments of risk of bias for each included study.	Quality of the Studies analyzed; Tables 6-7 (NIH/NHLBI ratings)
Results of individual studies	19	For all outcomes, present, for each study: (a) summary statistics for each group (where appropriate) and (b) an effect estimate and its precision (e.g. confidence/credible interval), ideally using structured tables or plots.	Results; Tables 1-3 (dose, BED10/EQD2, mPFS, mOS, LC, toxicity per study/arm)
Results of syntheses	20a	For each synthesis, briefly summarise the characteristics and risk of bias among contributing studies.	Results (Group A and Group B descriptions); Quality of the Studies analyzed; Tables 1-3, 6-7

PRISMA 2020 Checklist

Section and Topic	Item #	Checklist item	Location where item is reported
	20b	Present results of all statistical syntheses conducted. If meta-analysis was done, present for each the summary estimate and its precision (e.g. confidence/credible interval) and measures of statistical heterogeneity. If comparing groups, describe the direction of the effect.	Not applicable - no meta-analysis; results summarised narratively and in Tables 1-3
	20c	Present results of all investigations of possible causes of heterogeneity among study results.	Results (Group A vs Group B); Discussion (technical and clinical questions)
	20d	Present results of all sensitivity analyses conducted to assess the robustness of the synthesized results.	Not applicable - no sensitivity analysis performed
Reporting biases	21	Present assessments of risk of bias due to missing results (arising from reporting biases) for each synthesis assessed.	Not applicable - reporting bias in a synthesis not formally assessed
Certainty of evidence	22	Present assessments of certainty (or confidence) in the body of evidence for each outcome assessed.	Quality of the Studies analyzed; Tables 6-7; Limitations
**DISCUSSION**			
Discussion	23a	Provide a general interpretation of the results in the context of other evidence.	Discussion (paragraphs 1-2; Group A and Group B; Systemic therapy and the contemporary landscape)
	23b	Discuss any limitations of the evidence included in the review.	Discussion, Limitations sections
	23c	Discuss any limitations of the review processes used.	Discussion, Limitations (English-language and single-arm study restrictions)
	23d	Discuss implications of the results for practice, policy, and future research.	Discussion (Clinical and technical questions; ongoing trials); Conclusions
**OTHER INFORMATION**			
Registration and protocol	24a	Provide registration information for the review, including register name and registration number, or state that the review was not registered.	Material and Methods (last paragraph): registered in PROSPERO, CRD42024519535

PRISMA 2020 Checklist

Section and Topic	Item #	Checklist item	Location where item is reported
	24b	Indicate where the review protocol can be accessed, or state that a protocol was not prepared.	Material and Methods: PROSPERO (CRD42024519535)
	24c	Describe and explain any amendments to information provided at registration or in the protocol.	No amendments to the registered protocol
Support	25	Describe sources of financial or non-financial support for the review, and the role of the funders or sponsors in the review.	None
Competing interests	26	Declare any competing interests of review authors.	None
Availability of data, code and other materials	27	Report which of the following are publicly available and where they can be found: template data collection forms; data extracted from included studies; data used for all analyses; analytic code; any other materials used in the review.	Supplementary data (full search strategy); data extracted are available in Tables 1-7

From: Page MJ, McKenzie JE, Bossuyt PM, Boutron I, Hoffmann TC, Mulrow CD, et al. The PRISMA 2020 statement: an updated guideline for reporting systematic reviews. BMJ 2021;372:n71. doi: 10.1136/bmj.n71. This work is licensed under CC BY 4.0. To view a copy of this license, visit https://creativecommons.org/licenses/by/4.0/

Table S2. Study-level and arm-level audit of fatal (grade 5) toxicity in the 13 photon studies with contemporary simulation techniques (n = 644).

Study (trial)	N	Grade 5 events	Study-level grade 5	Fatal haemorrhage	Highest arm-/cohort-level grade 5
**GROUP A — non-concurrent strategies**					
Ohri et al. 2024 (SPRINT)	25	0	0% (0/25)	0	—
Parisi et al. 2019	17	0	0% (0/17)	0	—
Zhang W et al. 2015	28	0	0% (0/28)	0	—
Iyengar et al. 2021	96	NR	NR	NR	—
**Subtotal Group A**	**166**	**0**	**0% (0/70 evaluable)**	**0**	**—**
**GROUP B — concurrent platinum-based strategies**					
Kim et al. 2017	12	0	0% (0/12)	0	—
Urbanic et al. 2018 (CALGB 31102)	21	3	14.3% (3/21)	2/21 (9.5%)	33.3% (2/6), arm 3
Wu et al. 2024	28	2	7.1% (2/28)	2/9 (22.2%), highest cohort	22.2% (2/9), highest dose cohort
Qiu et al. 2021 (GASTO1011)	99	3	3.4% (3/89 boosted); 3.0% (3/99 enrolled)	2/89 (2.2%)	3.4% (3/89)
Van Diessen et al. 2020	47	NR	NR	NR	—
Cooke et al. 2023 (PET-Boost)	107	19	17.8% (19/107)	4/54 (7.4%) arm A; 3/53 (5.7%) arm B	20.8% (11/53), PET-subvolume arm

Roy et al. 2016	36	2	5.6% (2/36)	0	—
Glinski et al. 2020	92	5	5.4% (5/92)	≥2/92 (2.2%)	5.4% (5/92)
Katsuta et al. 2021	36	0	0% (0/36)	0	—
**Subtotal Group B**	**478**	**34**	**7.9% (34/431 evaluable); median 5.5% (range 0–17.8%)**	**≥15**	**33.3% (2/6)**
**TOTAL photon cohort**	**644**	**34**	**6.8% (34/501 evaluable)**	**≥15/644 (2.3%, 95% CI 1.4–3.8) in 5 studies**	**33.3% (2/6)**

Grade 5 = fatal toxicity (CTCAE). Study-level rates use the full study population as the denominator; where a fatal event occurred in a specific arm or dose cohort, the arm-level rate is given in the last column. Van Diessen et al. 2020 did not report toxicity by grade and Iyengar et al. 2021 reported grade ≥3 events in aggregate without separate grade 5 attribution; both studies are therefore excluded from the pooled denominators (501 evaluable patients overall, 431 in Group B). Confidence intervals are Wilson score intervals. NR = not reported; — = not applicable (no fatal event).
